# Prevention and Treatment of Cardiac Surgery Associated Acute Kidney Injury

**Published:** 2016-01

**Authors:** Hao Cheng, Jian-Zhong Sun, Fu-Hai Ji, Hong Liu

**Affiliations:** 1 Department of Anesthesiology, First Affiliated Hospital of Soochow University, Suzhou, China; 2 Department of Anesthesiology and Pain Medicine, University of California Davis Health System, Sacramento, USA; 3 Department of Anesthesiology, Thomas Jefferson University and Hospitals, Philadelphia, USA.

## Abstract

**Aim of review:**

Acute kidney injury (AKI) after cardiac surgery is a relatively common postoperative complication and is independently related to increased mortality and morbidity.

**Method:**

In this review, we will focus on risk factors of developing AKI, early detection by biomarkers and preventive strategies for AKI after adult cardiac surgery.

**Recent findings:**

Many perioperative factors affect renal function and acute AKI following cardiac surgery. Novel biomarkers of kidney injury such as neutrophil gelatinase-associated lipocalin (NGAL), interleukin-18 (IL-18), cystatin C (CysC), have the potential to facilitate the early diagnosis of cardiac surgery associated AKI (CSA-AKI). Pharmacological interventions have been inconsistent to their efficacy, and to date, there is no compelling pharmacologic agent known to reduce the risk of AKI or treat established AKI.

**Summary:**

Preventive strategies of AKI focus on optimal perioperative management.

Every year there are about 1,000,000 cardiac surgical procedures performed in the United States and Europe (1). Acute kidney injury (AKI) after cardiac surgery is a common postoperative complication and is independently associated with increased mortality, morbidity, and cost (2–10). AKI following cardiac surgery has a complex and multifactorial etiology. Patients with AKI requiring renal replacement therapies have mortality rates in excess of 40% to 50%. It is important that the identification of highrisk patients, development of protective strategies, and use of markers of kidney injury in the early detection and appropriate treatment of this serious complication. In this review, we will focus on risk factors of developing AKI, early detection by biomarkers and preventive strategies for AKI after cardiac surgery.

## Definition of AKI

Current diagnosis and staging of AKI are based on changes in serum creatinine (sCr) and urine output. The two most commonly used staging classifications are the Risk-Injury-Failure-Loss-End stage (RIFLE) (11) and the Acute Kidney Injury Network (AKIN) criteria (12). In 2004, the Acute Dialysis Quality Initiative (ADQI) Group established the RIFLE criteria. The RIFLE classification defines three grades of severity (risk, injury, and failure) and two outcome classes (loss of kidney function and end-stage kidney disease). The RIFLE uses a 7-day window (Table 1). The AKIN proposed a modification of the RIFLE classification in 2007. The AKIN classification uses a 48-hour time window. AKI by AKIN is defined as an increase in sCr levels of 0.3 mg/dl or greater, or an increase by 50% or more from baseline within 48 hours, or urine output less than 0.5 ml/kg/hour for more than 6 hours (Table 1). The AKIN system defines three progressive AKI stages without outcome classes. In this review, we will focus on occurrence, the risk factors, early detection and preventative strategy of AKI after adult cardiac surgery.

## Incidence of AKI

The true incidence of AKI is difficult to estimate because of the different definitions used in different studies. The reported incidence of AKI ranged from 1.1 to 57% (3–17), the variations are partially due to the different definitions of renal dysfunction used in each trial. The incidence of AKI can be influenced by many factors.

## Risk Factors of AKI

The pathogenesis of AKI after cardiac surgery is not completely understood, but is almost certainly multifactorial. Multivariate analyses have identified independent risk factors for cardiac surgery associated AKI (CSA-AKI). These risk factors can be divided into preoperative, intraoperative and postoperative factors.

### Preoperative Risk Factors

The occurrence of AKI within 7 days of cardiac surgery is usually due to preoperative and operative factors. The risk is influenced by demographic factors, comorbid conditions. Preoperative factors include advanced age, female gender, anemia, a large delta mean arterial pressure (MAP), preexisting isolated systolic hypertension, pulse pressure >40 mm Hg, chronic kidney disease, low ejection fraction, congestive heart failure, diabetes mellitus, peripheral vascular disease, chronic obstructive pulmonary disease, use of intra-aortic balloon pump (IABP), emergency surgery, and elevated sCr (2, 15, 16, 18, 21).

### Intraoperative Risk Factors

Intraoperative risk factors include the aorta cross-clamped, cross-lamp time, intraoperative systolic blood pressure (SBP), hypotension, excursions of MAP beyond the limit of autoregulation, cardiopulmonary bypass (CPB), lower CPB flow, duration of CPB, use of inotropes, mitral valve surgery, reoperation, blood transfusion and reinitiation of CPB (2, 15, 16, 19–21).

### Postoperative Risk Factors

AKI, occurs between 7 and 30 days postoperatively, can often be related to postoperative factors such as low cardiac output or sepsis, erythrocytes transfusion, administration of vasoconstrictors, inotropes, diuretics, and antiarrhythmic medications (15, 21).

### Anemia and Transfusion

Studies demonstrated that preoperative anemia, intraoperative anemia, and red blood cell (RBC) transfusion on the day of surgery were all significantly associated with AKI during cardiac surgery (23). The occurrence of AKI was significantly increased when intraoperative hemoglobin decreased more than 50% below baseline (24). Parolari and colleagues (15) indicated that intraoperative and postoperative RBC transfusion was significantly associated with AKI development. Karkouti (25) noted that perioperative transfusion and anemia likely have a synergistic effect on the risk of AKI. A recent study by Haase and colleagues (26) indicated that decreased hemoglobin concentration with an effect cut-off value of < 9 g/dl and volume of transfused RBC were an independent risk factor for AKI. Avoiding severe hemodilution and transfusion in patients with hemoglobin levels > 8 g/dl may help decrease AKI development in patients undergoing cardiac surgery (26).

There are several therapeutic options that can help avoid or reduce the potential harms of perioperative RBC transfusions on the kidney following cardiac surgery with CPB. The first option is to reduce the rate of perioperative transfusions. The second is the prophylactic transfusions 1–2 days before surgery in patients with preoperative anemia (27). The third treatment is to use erythropoietin-stimulating agents to reduce the need for perioperative transfusions in anemic patients (28). The fourth strategy is to improve the quality of the transfused blood for reducing its harmful effects (25).

### Perioperative Blood Pressure

Recent data suggested that preexisting isolated systolic hypertension and blood pressure outside an acceptable physiologic range during cardiac surgery were associated with increased risk for AKI (18). Intraoperative SBP decrease relative to baseline SBP is independently associated with postoperative AKI in coronary artery bypass graft (CABG) surgery patients (19). Another study suggested that a higher magnitude and duration of MAP below the lower limit of cerebral autoregulation was independently associated with AKI (20). A large delta MAP (the preoperative MAP minus average intraoperative MAP) during cardiac surgery is independently associated with early post-operative CSAAKI in high-risk patients (16). Aronson and colleagues noted that preoperative pulse pressure >40 mm Hg and MAP <60 mm Hg during CPB to be significantly associated with the development of CSA-AKI.

### Cardiopulmonary Bypass

A meta-analysis of 46 studies with 47 unique cohorts comprising 242,388 participants showed that the pooled rate of CPB associated AKI was 18.2%, and CPB-relate AKI is associated with a more than 2-fold increase in early mortality (5). CPB itself contributes to the pathogenesis of AKI by activating a systemic inflammatory response, altering regional blood flow and vasomotor tone in kidneys and generating microemboli. The mechanical destruction of erythrocytes causes release of plasma free hemoglobin into the circulation, which causes occlusion of renal tubules and necrosis of tubular cells (29). CPB related embolization that may be composed of fibrin, platelet aggregates, cellular debris, fat and air, is associated with the occurrence of AKI (30). A recent study of 1,185 patients found that the pulsatile perfusion had significantly higher creatinine clearance (P=0.004) and lower serum lactate levels (P=0.012) (31), and it showed that pulsatile perfusion during CPB might be beneficial in renal preservation. A target MAP of at least 60 mm Hg during CPB may reduce the risk of AKI after CPB (32). A prospective observational study of 157 patients indicated that lower CPB flow during cardiac surgery was independently associated with early postoperative CSA-AKI in high-risk patients (16). The duration of CPB is another independent risk factor for AKI in the postoperative setting (22, 33). At present, there is no single defined threshold time during CPB beyond which the incidence of AKI increases dramatically. The relationship between the nadir hematocrit (Hct) value during CPB and AKI was confirmed in a multivariable analysis, with the relative risk of AKI increasing by 7% per percentage point of decrease of the nadir Hct value during CPB.

### Types of Surgery

Among the different types of surgery, isolated CABG has the lowest incidence of AKI followed by valve surgery and combined CABG with valve surgery. One study of 9,222 patients reported that the overall incidence of severe AKI was 1.2%, but it differed with the types of surgical procedures including CABG surgery, 0.4% ; heart valves, 1.7% ; aorta surgery, 5.4% ; ventricle septum rupture, 52.6% ; and other, 6.5% (9). Arnaoutakis and colleagues reported that 48% of patients had an episode of AKI in aortic arch surgery with deep hypothermic circulatory arrest. A retrospective analysis showed the incidence of AKI was 44.0% using the RIFLE criteria after total aortic arch repair with moderate hypothermic circulatory arrest. It has been reported that AKI incidence was 3.4–43% in open-heart aortic valve replacement cases, and 3.4–57% in transcatheter aortic valve implantation (TAVR) cases (6, 7).

Off-pump CABG surgery is substantially different from on-pump CABG surgery, avoiding cannulation and cross-clamping of the aorta and cardioplegic arrest. Thus, off-pump CABG appears to be a logical step toward minimizing the risk of postoperative AKI. However, studies have provided conflicting results (17, 34–37). A randomized, controlled, multicenter trial with 4, 752 patients suggested that although the use of off-pump CABG resulted in reduced rates of AKI (RR 0.87; 95 % CI 0.76–0.98, P=0.02), there was no significant difference in the 30-day mortality rate, myocardial infarction, stroke and renal failure requiring dialysis (34). The CORONARY trial at one year follow-up found that off-pump compared with on-pump CABG surgery reduced the risk of postoperative AKI, but did not alter one year kidney function (35). Whereas, other studies did not support the assumption that off-pump CABG could decrease the risk of postoperative AKI (17, 36, 37). Although the postoperative glomerular filtration rate (GFR) decreases significantly after CABG, there was no difference in occurrence of postoperative AKI between on-pump and off-pump CABG (17, 37). These conflicting results may be due to the multiple risk factors that predispose to the development of postoperative AKI.

### Blood Glucose Level

A retrospective analysis on 1,050 patients showed that maintaining perioperative blood glucose levels between 80 and 110 mg/dl could be associated with a significant reduction in AKI in non-diabetic patients undergoing cardiac surgery (38). However, Nice-Sugar study, a large randomized clinical trial, demonstrated no benefit in tight glucose control that targeted a blood sugar level of <108 mg/dl and had an unacceptable incidence of hypoglycemia in critically ill patients (39).

### Contrast Agent Exposure

There is an increased use of contract agents in patients undergoing cardiac surgery recently. Contrast agents induced nephropathy is one of the common complications after cardioangiography (40). Zhang and colleagues (41) showed that a selective off-pump CABG within 24 hours after coronary angiography could significantly increase the risk of postoperative AKI and suggested that selective off-pump CABG, if possible, should be performed >24 hours after coronary angiography. Medalion et al. (42) suggested that CABG should be delayed for at least 5 days in patients who received a high contrast dose, especially if they also have preoperative reduced renal function. However, other studies have demonstrated that the preoperative contrast is not associated with postoperative AKI (43, 44).

## Early Diagnosis of AKI

Diagnosis of AKI is mainly based on the increases in sCr that indicates the loss of excretory renal function. sCr is an insensitive and unreliable biomarker during short-term changes in kidney function because sCr requires hours to days to accumulate. Moreover, sCr is also affected by age, race, muscle mass, volume of distribution, medications and protein intake (45). It does not discriminate the nature of ischemic and pre-renal insult. Changes in sCr occurred late in the development of AKI, typically 48 hours after the initiating event. Hence, its levels may remain normal despite significant AKI. Despite its limitations, sCr remains an important outcome biomarker in cardiac surgery. Subclinical increases in sCr that do not meet AKI criteria are independently associated with 30-day all-cause mortality in patients with normal renal function or preoperative renal insufficiency undergoing CABG (36).

However, there is a need for more sensitive and specific biomarkers that can diagnose AKI earlier, possibly indicate the cause, stratify risk, identify AKI subtypes, and rapidly measure the response to therapy.

### Neutrophil Gelatinase-Associated Lipocalin

Human neutrophil gelatinase-associated lipocalin (NGAL) has been identified in neutrophils and proximal renal tubular epithelium. Urine NGAL is elevated as early as 2 hours after CPB, so it is an early predictive biomarker of AKI after cardiac surgery (46–48). McIlroy and colleagues (49) reported that elevated postoperative serum NGAL best identified AKI in patients with baseline estimated GFR (eGFR) 90 to 120 ml/minute. Urinary NGAL was considered superior to plasma NGAL and sCr in the early diagnosis of CSA-AKI (50). Koyner and colleagues (14) thought that plasma NGAL had the strongest ability to predict the progression of AKI, but urine NGAL measurement has been found less beneficial after adjusting for variables known to impact AKI risk. Additionally, plasma NGAL did not correlate well with the urinary biomarkers. Others found that urine and plasma NGAL levels peaked within 6 hours after surgery, and they concluded that urine and plasma NGAL associated with subsequent AKI and poor outcomes among adults undergoing cardiac surgery (48). Lipcsey and colleagues (51) suggested that plasma NGAL acts as a neutrophil activation biomarker and urinary NGAL as a tubular injury marker. A clinic study of 75 patients also showed that serum NGAL is not a stable predictive biomarker for AKI after on-pump CABG surgery (52).

### Cystatin C

Cystatin C (CysC) is an endogenous cysteine proteinase inhibitor with a molecular weight of 13 kDa. Serum CysC levels can be detected as early as 2–4 hours after cardiac surgery. It is more sensitive for reduced GFR than kidney Injury. In a cohort of 1,147 adult cardiac surgery patients, preoperative serum CysC performed better than creatinine or creatinine-based eGFR at forecasting the risk of AKI (53). Demirtas and colleagues reported that increased urinary CysC excretion is a useful early biomarker of AKI in adults following cardiac surgery, and the extent of excretion of this marker correlates with the severity of AKI, whereas plasma CysC was not a useful predictor of AKI within the first 6 hours following surgery (50).

### Interleukin-18

Interleukin-18 (IL-18) is a pro-inflammatory protein, which mediates ischemic tubular injury, and has been suggested to be a sensitive and specific biomarker for AKI (54). IL-18 is specific to the renal tubules and is known to be up-regulated in response to ischemia/reperfusion injury (55). Urine IL-18 starts to increase at 4–6 hours after CPB, peaks at over 25-fold at 12 hours and remains markedly elevated up to 48 hours after CPB (47). A prospective, multicenter cohort study involving 1,219 adult cardiac surgery patients concluded that urine IL-18 was associated with subsequent AKI and poor outcomes among adults undergoing cardiac surgery (48).

Early diagnosis of AKI by using new serum and urinary biomarkers remains controversial in clinical practice. Moreover, it is unlikely that a single biomarker will fulfill all identifications. Further research in this field is needed to confirm the early valid biomarkers that can identify early AKI and test whether earlier intervention favorably influences renal function and overall perioperative outcomes in clinical practice.

## Strategies to Prevent AKI

Several strategies and techniques have been used to prevent acute renal injury during cardiac procedures. Pharmacological interventions have been inconsistent to their efficacy, and there is no drug that has been convincingly and widely accepted for renal protection at present. Several preventive strategies focusing on preoperative, intraoperative and postoperative management are discussed here.

### Pharmacological Methods

#### Erythropoietin

It has been suggested a renal protective effect of erythropoietin through attenuation of polymor-phonuclear leukocytes that decreases systemic low-grade inflammation and oxidative stress (56). One study in elective CABG patients found that the infusion of erythropoietin (300 IU/kg) before surgery reduced the risk of AKI and improved postoperative renal function (57). Another study of single-dose erythropoietin (500 IU/kg) plus an iron supplement given 1 day before surgery showed a significant reduction in incidence of AKI (58). However, Dardashti and colleagues (59) found that infusion of a single bolus erythropoietin (400 IU/kg) after anesthetic induction did not have renal protective effect on patients with reduced kidney function undergoing on-pump CABG surgery. Kim and colleagues (60) found that intravenous use of a bolus 300 IU/kg of erythropoietin after anesthetic induction did not decrease the risk of developing AKI after undergoing complex valvular heart surgery. Therefore, erythropoietin infusion could reduce the incidence of AKI, but not in the patients with high risk factors for AKI.

#### Statins

Statins are known to attenuate inflammation and improve endothelial dysfunction in addition to the cholesterol-lowering efficacy. A study by Singh et al. (61) found that preoperative statin therapy resulted in a significantly lower renal replacement therapy (RRT) in patients undergoing CABG only, not the incidence of AKI. Other retrospective investigations indicated that preoperative statin therapy also reduced postoperative mortality, atrial fibrillation and stroke, but did not reduce the incidence of postoperative AKI (62–64). A double-blind randomized controlled trial in 100 cardiac patients also failed to validate statin treatment for 4 days starting preoperatively as a means of reducing the incidence of AKI after cardiac procedure (65). It is noted that statin use within the first postoperative day is associated with a lower incidence of AKI among both chronic statin users and statin-naive cardiac surgery patients (66).

#### Diuretics

In a prospective randomized clinical trial of patients undergoing cardiac surgery, prophylactic infusion of furosemide during the operation and after operation was associated with an increased incidence of AKI and deterioration of renal function (67). Similarly, a prospective randomized clinical trial of high-risk patients undergoing cardiac surgery did not demonstrate any benefit of prophylactic diuretic use to prevent AKI (68). Although urinary output increased with furosemide, routine postoperative diuretic administration has not been shown to prevent AKI or offer renal protection in CABG surgery (69). Therefore, diuretics remain a valuable tool for correcting volume overload, but they are not recommended for the prevention or treatment of AKI.

#### Sodium Bicarbonate

Urinary alkalinization in patients at risk of AKI undergoing cardiac surgery remains an important and controversial issue. A single-center randomized controlled pilot trial with 100 patients reported a statistically significant reduction in the incidence of AKI (P<0.043) after perioperative sodium bicarbonate use in patients undergoing cardiac surgery (70). A meta-analysis by Bailey et al. (71) suggested that urinary alkalinization using sodium bicarbonate in cardiac surgery patients did not significantly reduce the overall risk of AKI. However, it reduced severe AKI rate and need for RRT in elective CABG patients. Another prospective observational cohort study of cardiac surgical patients concluded that routine perioperative infusion of 4 mmol/kg sodium bicarbonate failed to improve postoperative renal function (72). Furthermore, a recent multicenter, double-blinded, randomized controlled trial on 350 high-risk patients for developing AKI showed that more patients received bicarbonate developed AKI compared with patients in the control group (47.7% vs. 36.4%, odds ratio [OR] 1.60, 95% confidence interval [CI] 1.04–2.45, unadjusted P=0.032) (73). The debate is still ongoing, but current data do not actually support routine use of bicarbonate to reduce the risk of AKI.

#### Angiotensin-Converting Enzyme Inhibitors and Angiotensin II Receptor Blockers

Most patients who undergo cardiac surgery receive long-term treatment with angiotensin-converting enzyme inhibitors (ACEI) or angiotensin II receptor blockers (ARB). Whether ACEI and ARB should be discontinued before surgery is still controversial (74–76). One study by Arora et al. (74) found that the preoperative use of ACEI or ARB was associated with a 27.6% higher risk for AKI after cardiac surgery and stopping ACEI or ARB before cardiac surgery may reduce the incidence of AKI. In contrast, a large prospective observational study in patients undergoing CABG (N=4,224) showed that continuous treatment of ACEI versus withdrawal of ACEI was associated with decreased risk of the composite outcome (OR, 0.50; 95% CI, 0.38–0.66; P<0.001), as well as a decrease in cardiac and renal events (P<0.001 and P=0.005, respectively) (75). A retrospective observational study found that preoperative ACEI decreased the incidence of postoperative AKI after on-pump CABG (76). Yoo and colleagues (77) reported that preoperative use of ACEI did not affect postoperative renal function or increase the risk of postoperative AKI after off-pump CABG. In brief, perioperative studies of the effects of ACEI remain limited and inconclusive. Results from recent clinical trials and observational studies are conflicting and raise more questions than answers. Further studies, both retrospective and larger-scale prospective studies, are critically needed to examine whether ACEI reduce mortality and major complications in patients undergoing cardiac surgery (78).

#### Fenoldopam

Fenoldopam is a selective dopamine receptor D1 agonist, which induces vasodilation of the renal, peripheral, and coronary arteries (79). Recent study of 440 patients showed that fenoldopam significantly reduced the risk of CSA-AKI (OR, 0.41, 95% CI, 0.23–0.74; P=0.003) with no effect on RRT requirement and mortality (80). A meta-analysis suggested that fenoldopam, increasing renal blood flow in a dose-dependent manner, has been observed to reduce AKI after cardiac surgery (81, 82). A multicenter, randomized, double-blind, placebo-controlled, parallel-group study showed that fenoldopam infusion did not reduce the need for RRT or risk of 30-day mortality among patients with AKI after cardiac surgery, but was associated with an increased rate of hypotension (83).

#### Nesiritide

Nesiritide is the recombinant human type B natriuretic peptide (BNP). A multicenter randomized controlled trial found that infusion of nesiritide to patients with left ventricle (LV) dysfunction undergoing CABG with CPB improved postoperative renal function, reduced hospital length of stay (LOS) and decreased 6 month mortality rate (84). The prospective, randomized, clinical trial included 94 patients undergoing high-risk cardiac surgery indicated that prophylactic use of nesiritide for 5 days before surgery reduced occurrence of AKI compared with control group (2.2% vs. 22.4% ; P=0.004), but had no effect on incidence of dialysis and/or allcause mortality through day 21 (P=0.914) (85). Based on above evidence, natriuretic peptides cannot be recommended for routine use in the patients with the risk of AKI who are undergoing cardiac surgery.

#### Aspirin

It is common that cardiac surgery patients take aspirin. An observational cohort study of 4,256 patients suggested that preoperative aspirin treatment was associated with a significant decrease in the risk for postoperative renal failure (OR, 0.384, 95% CI, 0.254–0.579, P<0.001) and 30-day mortality (OR, 0.611, 95% CI, 0.391–0.956, P=0.031) (86). Another retrospective cohort study of 3,585 patients indicated that preoperative aspirin therapy was associated with renal protection and mortality decline for patients with chronic kidney disease undergoing cardiac surgery (87). Recently, A single-center retrospective cohort analysis of 9,903 consecutive patients undergoing cardiac operations showed that preoperative aspirin use was associated with lower risks of postoperative renal failure (OR, 0.91) in CABG surgery, and was not statistically significantly different in valve operations (88). A 2 × 2 factorial randomized, blinded, clinical trial of 6, 905 patients demonstrated that perioperative aspirin administration did not reduce the risk of AKI (adjusted relative risk, 1.10; 95% CI, 0.96–1.25) among patients undergoing major noncardiac surgery (89). Further investigations, especially large-scale, randomized, and controlled trials, will be needed to confirm that the use of aspirin reduces the AKI rate in cardiac surgery patients.

Overall, there is no single pharmacologic agent that has been unquestionably demonstrated to reduce the risk of AKI or treat established AKI at this time.

### Non-Pharmacological Strategies

In the preoperative period, the major goals include improving cardiac function, optimizing renal function, avoiding intravascular volume depletion and avoiding anemia (23, 90). In the intraoperative period, optimal CPB is vital that include using pulsatile perfusion, avoiding severe hemodilution and packed RBC transfusion, and keeping MAP>60 mm Hg (22, 26, 31–33). Minimally invasive surgery might be also renoprotective (91, 92). In the postoperative period, the early use of RRT (<3 days after cardiac surgery) may be an important factor used to increase survival in patients with CSA-AKI (93). Strategies with a decreased risk of AKI after cardiac surgery are summarized in Table 2.

It is well known that most risk factors for postoperative AKI are not and/or difficult to be modifiable, such as advanced age, gender, diabetes, poor heart function, preoperative renal insufficiency and emergency surgery. It is particularly important to identify risk factors that can be modified or avoided. However, up to now, many approaches are often controversial due to the complexity of AKI pathophysiology. There is little compelling evidence from randomized trials supporting specific interventions to protect or prevent AKI after cardiac surgery. However, it is important to improve perioperative hemodynamic management during cardiac surgery.

## Conclusions

AKI following cardiac surgery is a common postoperative complication that is associated with increased morbidity, mortality and costs. It is worth noting the value of NGAL, CysC and IL-18 in early diagnosis of AKI following cardiac surgery. Due to the complex pathogenesis of AKI, it is unlikely that any one of the interventions can reduce incidence of AKI following cardiac surgery, thus an integrated comprehensive approach of preventions and treatments of AKI needs to be considered. Current strategies to prevent AKI after cardiac surgery include: avoiding nephrotoxins, controlling blood glucose, maintaining perioperative hemodynamic optimization, blood transfusing and inotropic support, considering of use less invasive procedures, avoiding prolonged aortic cross-clamping and CPB, and initiating RRT early for patients with CAS-AKI.

## Figures and Tables

**Table 1.** Comparison of RIFLE and AKIN Criteria for Acute Kidney Injury.

	RIFLE criteria (within 7 days)			AKIN (within 48 hours)	
Class	GFR or Creatinine	Urine output	Stage	Creatinine	Urine output
Risk	GFR decrease >25%, or increase 1.5 times (baseline)	<0.5 ml/kg/hour for >6 hours	1	Cr 1.5 times (baseline), or increase of >0.3 mg/dl	<0.5 ml/kg/hour for >6 hours
Injury	GFR decrease >50% or Cr increase 2 times (baseline)	<0.5 ml/kg/hour for >12 hours	2	Cr 2 times (baseline)	<0.5 ml/kg/hour for >12 hours
Failure	GFR decrease >75% or Cr increase 3 times (baseline), or Cr >4 mg/dl (acute increase > 0.5 mg/dl)	<0.3 ml/kg/hour for >24 hours or anuria >12 hours	3	Cr 3 times (baseline), or > 4 mg/dl (acute increase > 0.5 mg/dl) or RRT	<0.3 ml/kg/hour for >24 hours or anuria >12 hours
Loss	Persistent ARF=Complete loss of renal function >4 weeks				
ESRD	Complete loss of renal function without recovery >3 monthst				

RIFLE: Risk-Injury-Failure-Loss-End stage kidney disease; AKIN: Acute Kidney Injury Network; ARF: acute renal failure; Cr: creatinine; RRT: renal replacement therapy; ESRD: end-stage renal disease; GFR: glomerular filtration rate.

**Table 2.** Strategies with a Decreased Risk of AKI after Cardiac Surgery.

Preoperative	Intraoperative	Postoperative
Avoid diuretics	Optimal glucose control	Start statin when feasible
Prudent blood transfusion	Avoid red blood cells transfusion	Use of early (<3 days) RRT
Optimal SBP	MAP>60 mm Hg during CPB	
Surgery after 1–5 days of angiography	Shorter duration of CPB Miniature CPB systems Minimally invasive surgery	
Aspirin in CABG	Avoid serve hemodilution Pulsatile perfusion	

AKI: acute kidney injury; RRT: renal replacement therapy; SBP: systolic blood pressure; MAP: mean arterial pressure; CPB: cardiopulmonary bypass; CABG: coronary artery bypass graft.
